# Assessment of Sling Characteristics After Transobturator Tape Placement Using Transperineal Ultrasonography: Relationship with Continence and Dyspareunia—A Cross-Sectional Study

**DOI:** 10.3390/jcm15197613

**Published:** 2026-10-01

**Authors:** Mehmet Koçoğlu, Oya Soylu Karapınar, Muhammed Tosun, Berna Haliloğlu Peker

**Affiliations:** 1 Department of Obstetrics and Gynecology, Tayfur Ata Sökmen Faculty of Medicine, Hatay Mustafa Kemal University, 31000 Antakya, Turkey; 2 Van Provincial Health Directorate, 65100 Van, Turkey; 3 Division of Urogynecology, Department of Obstetrics and Gynecology, Maltepe University Faculty of Medicine Hospital, 34857 Istanbul, Turkey

**Keywords:** stress urinary incontinence, transobturator tape, transperineal ultrasound, midurethral sling, dyspareunia, surgical success

## Abstract

**Background/Objectives**: Stress urinary incontinence (SUI) is a common condition that adversely affects women’s quality of life, and transobturator tape (TOT) surgery is one of the most commonly used treatments; however, the determinants of surgical success and postoperative complications remain incompletely understood. The aim of this study was to evaluate the association between postoperative transperineal ultrasound (TPUS) findings and surgical outcomes after TOT surgery for SUI, with particular emphasis on the relationship between sling arm morphology and postoperative dyspareunia. **Methods**: In this single-centre, cross-sectional follow-up study of a retrospectively identified cohort, 74 of 148 women who underwent TOT surgery between February 2019 and November 2024 were evaluated. TPUS was used to assess sling position, morphology, mobility, deformability, symphysis pubis–sling distance (SP-TAPE), urethral concordance, and bladder wall thickness. Patient-reported outcomes were evaluated using the I-QOL, UDI-6, IIQ-7, and OAB-V8 questionnaires. Factors independently associated with surgical success were identified using multivariable logistic regression analysis. **Results**: Objective surgical success was achieved in 71.6% of patients, whereas 91.9% reported satisfaction with the outcome. Concordant movement of the sling with the urethra (OR 8.963, 95% CI 1.612–49.857, *p* = 0.012), a shorter SP-TAPE distance during Valsalva (OR 0.337, 95% CI 0.181–0.629, *p* = 0.001) and a thinner bladder wall (OR 0.375, 95% CI 0.185–0.759, *p* = 0.006) were independently associated with objective success. Although sling arm morphology was not associated with surgical success (*p* = 0.797), among sexually active women (*n* = 61), curved sling arms were strongly associated with postoperative dyspareunia (75.0% vs. 27.3%; OR 8.00, 95% CI 2.54–25.22, *p* < 0.001). Bladder wall thickness correlated moderately with all four instruments (|r| = 0.551–0.667; all *p* < 0.001), inversely with I-QOL and positively with UDI-6, IIQ-7, and OAB-V8. **Conclusions**: Successful TOT surgery depends not only on appropriate sling localization but also on concordant sling–urethral movement and favourable dynamic sling behaviour. Although sling arm morphology was not associated with continence outcomes, its association with postoperative dyspareunia highlights the importance of evaluating both functional cure and postoperative quality of life. In the absence of preoperative data, this association should be regarded as exploratory. TPUS provides clinically valuable information for the postoperative assessment of outside-in transobturator slings; because all assessments were performed at a single visit, the relationships reported here are associations rather than demonstrated predictive relationships.

## 1. Introduction

Stress urinary incontinence (SUI) is defined as the involuntary loss of urine occurring with increases in intra-abdominal pressure and is the most common type of urinary incontinence in women [1,2]. Affecting approximately one-third of women worldwide, it markedly impairs quality of life by adversely affecting social life, physical activity, working life, and sexual function [3]. Although the reported prevalence in Turkish women ranges from 16.4% to 49.7%, many women regard urinary leakage as a natural consequence of ageing and do not seek medical help. SUI therefore remains an important public health problem in which diagnosis and treatment are frequently delayed [4].

Pelvic organ prolapse (POP) and SUI are the two most frequent pelvic floor disorders and together affect a substantial proportion of adult women, diminishing quality of life and affecting social functioning, working life, and mental health [2,3,4]. Although they frequently coexist, they arise from partly distinct mechanisms and require different diagnostic and therapeutic approaches; identifying the predominant dysfunction is therefore an essential step in clinical decision-making [2]. Transperineal ultrasound is well suited to this task, since it allows both the anatomy and the dynamic behaviour of the anterior compartment to be assessed within a single examination [5].

Transobturator tape (TOT) surgery is currently one of the most widely performed procedures in SUI surgery, with reported objective success rates of 80–90% [6,7]. Nevertheless, persistent or recurrent incontinence may develop in 5–23% of patients, and complications such as de novo overactive bladder, obstructive voiding, dyspareunia, and pelvic pain may occur even in cases considered objectively successful [8,9,10]. These complications adversely affect quality of life and sexual function, indicating that objective surgical success does not always coincide with functional outcomes that are satisfactory from the patient’s perspective. Moreover, the mechanisms determining surgical success and postoperative complications have not yet been fully elucidated.

The mechanism of action of midurethral slings remains a matter of debate. One view holds that surgical success is related to the static position of the sling and its dynamic behaviour during the Valsalva manoeuvre [11], whereas the other argues that the fibrotic reaction developing in the surrounding tissue is the principal determinant [12]. Transperineal ultrasound (TPUS) offers considerable advantages in clarifying this debate owing to its non-invasive nature and its capacity for real-time imaging [5,13]. Although previous studies have shown that sling location, urethral concordance, and sling deformability are associated with surgical success [8,14,15], most of these studies focused solely on comparing successful and unsuccessful surgical outcomes. In contrast, the relationship between sling arm morphology and postoperative dyspareunia, the link between bladder wall thickness and symptom burden, and the ultrasonographic markers of postoperative overactive bladder have not been sufficiently investigated.

The aim of this study was to provide a comprehensive evaluation of the relationship between the static and dynamic characteristics of the sling assessed by transperineal ultrasound and both surgical success and postoperative functional outcomes in women who had undergone TOT surgery. In addition to surgical success, ultrasonographic markers of postoperative complications such as dyspareunia, urge urinary incontinence, and pelvic pain were investigated. Unlike previous studies, this investigation evaluated not only sling location but also sling arm morphology and dynamic sling behaviour together. The aim was thus to demonstrate the relationship of ultrasonographic findings after TOT surgery with both surgical success and patient-centred functional outcomes and to examine the potential contribution of these parameters to clinical assessment.

## 2. Materials and Methods

### 2.1. Study Design and Ethical Approval

This single-centre, cross-sectional follow-up study comprised patients who had undergone outside-in transobturator tape (TOT) surgery for stress urinary incontinence (SUI) at the Department of Obstetrics and Gynecology, Tayfur Ata Sökmen Faculty of Medicine, Hatay Mustafa Kemal University. Patients were identified retrospectively from hospital records, whereas transperineal ultrasound, physical examination, and questionnaire assessments were performed prospectively at a single follow-up visit within the scope of the study. The study was reported in accordance with the STROBE guideline for cross-sectional studies [16] (Table S1). The research was approved by the institutional Non-Interventional Research Ethics Committee (17 September 2025, Decision No: 31) and was conducted in accordance with the principles of the Declaration of Helsinki. Following ethics committee approval, patients who had undergone TOT surgery between February 2019 and November 2024 were identified from hospital records and invited for a follow-up examination; written informed consent was obtained from all participating patients.

The primary endpoint of the study was to characterise the relationship between the static and dynamic sling features detected by transperineal ultrasound and postoperative surgical success and complications (dyspareunia, pelvic pain, postoperative OAB); the relationship between sling arm morphology and dyspareunia was the principal focus of the study.

### 2.2. Participants and Sample

Patients aged 18–75 years who had undergone TOT surgery for SUI at our clinic and had completed at least 12 months of postoperative follow-up were included. Exclusion criteria were pelvic organ prolapse (POP) of POP-Q stage 2 or above detected at the postoperative assessment, major psychiatric or neurological disease, pregnancy or the first six postpartum months, active urogenital or pelvic infection, pelvic inflammatory disease, uncontrolled diabetes mellitus, collagen tissue disease, and acute gynaecological pathology. Concomitant anterior compartment repair was performed in 12 of the 74 patients (16.2%); these patients were not excluded. Likewise, patients who developed postoperative overactive bladder, obstructive voiding or other complications were not excluded; the ultrasonographic characteristics of these cases were evaluated separately. All procedures were performed by the same single surgeon, a professor of obstetrics and gynaecology, assisted by resident physicians, using a standardised outside-in transobturator technique; this arrangement was planned at the outset of the study in order to eliminate inter-surgeon variability in sling placement. The same sling material was used in every patient: a monofilament polypropylene tape measuring 10 × 500 mm with a pore size of ≥50 μm, corresponding to a type I macroporous mesh (SuproSui, Klas Medikal, Istanbul, Türkiye).

Because of the retrospective design, the sample size was limited to the available archival records, and no a priori power analysis was performed. Of the 148 patients screened, 53 could not be contacted, 19 declined to participate, and 2 were excluded because of exclusion criteria; the remaining 74 patients were included in the analysis (Figure 1).

### 2.3. Clinical Assessment and Instruments

At the follow-up examination, age, gravidity, parity, mode of delivery, menopausal status, body mass index (BMI), smoking and alcohol use, sexual activity, chronic diseases, previous surgery, and time since surgery (months) were recorded. Patients were questioned dichotomously (yes/no) regarding stress urinary incontinence, postoperative urge incontinence/overactive bladder (OAB), dyspareunia, and pelvic pain. Because preoperative records of these symptoms were not available, patients were asked whether the symptom had begun after surgery; the postoperative onset of urge incontinence/OAB therefore rests on patient report rather than on documented preoperative assessment. In accordance with International Continence Society definitions, objective surgical success was defined as the absence of urinary leakage on the stress test described in Section 2.4 [2].

Four instruments with demonstrated validity and reliability in Turkish were administered: I-QOL [17,18], UDI-6 [19,20], IIQ-7 [19,20] and OAB-V8 [21,22]. On the UDI-6, IIQ-7, and OAB-V8, higher scores indicate a greater symptom burden, whereas on the I-QOL, a higher score indicates better quality of life. Patient satisfaction was assessed with a three-category Likert scale: (1) not satisfied, (2) satisfied, and (3) very satisfied. For the logistic regression analysis, patients were grouped as satisfied (2–3) and not satisfied (1). The questionnaires were administered face-to-face by a senior resident who was not involved in the surgery or in the ultrasonographic assessment.

### 2.4. Physical Examination and Ultrasonography

Physical examination and questionnaire administration were performed by a physician not involved in the surgery. Patients were asked to refrain from voiding for two hours; clinical SUI was then assessed with a cough stress test at full bladder filling, after which patients voided, and the post-void residual volume was measured using the standard ellipsoid formula. All subsequent ultrasonographic measurements were obtained at this post-void residual volume, which averaged 82.1 ± 20.7 mL (range 45–136 mL). Vaginal examination was performed to assess pelvic organ prolapse and mesh extrusion. All ultrasonographic assessments were performed by a single investigator with three years of experience in ultrasonography who had completed a one-month dedicated training programme in pelvic floor ultrasound under the supervision of an expert in the field, using a Samsung HS50 system (Samsung Medison Co., Ltd., Seoul, Republic of Korea) and a convex probe (CA1-7AD, 1–7 MHz); this physician was blinded to the clinical, questionnaire, and examination findings of the patients.

Patients were assessed in the dorsal lithotomy position with a bladder volume below 200 mL. The probe was directed from the interlabial region towards the symphysis pubis, and the bladder, urethra, and symphysis pubis were visualised in the transsagittal plane; probe rotation was avoided in order to prevent anatomical distortion. A maximal Valsalva manoeuvre of at least six seconds was performed to prevent levator ani co-activation; at least two successful recordings were obtained in every patient, and the manoeuvre was repeated when the difference exceeded 10%.

### 2.5. Ultrasonographic Parameters

The sling was identified as a hyperechoic structure dorsal to the urethra [23]. The urethra was divided into three equal segments (proximal, mid, and distal), and sling location was recorded at rest and during Valsalva. Movement of the sling together with the urethra during Valsalva was defined as concordant, and independent movement as discordant [14]. Sling morphology was classified into four categories (straight, S, C, and other); a change in shape between rest and Valsalva was considered ‘deformable’. The S-shape category extends the three-category system of Hegde et al. [14]. In the transverse plane, arm morphology was classified according to the course of the sling arms as a straight course (‘non-curved’) or a curved course (‘curved’); curvature of the arms was the sole criterion applied, and the symmetry of the two arms was not used for classification (Figure 2a,b).

The relationship of both parameters with surgical success and complications (pelvic pain, dyspareunia) was analysed separately. In addition, bladder neck position relative to the inferior margin of the symphysis pubis was measured separately at rest and during Valsalva, and bladder neck descent (BND) was derived as the difference between the two; the minimum symphysis–sling distance (SP-TAPE), post-void residual volume (standard ellipsoid formula), and bladder wall thickness were measured. Bladder wall thickness was measured at the anterior bladder wall as a single measurement under the filling conditions described in Section 2.4.

### 2.6. Statistical Analysis

Analyses were performed with SPSS v31.0 (IBM Corp., Armonk, NY, USA). Categorical variables are presented as numbers/percentages and continuous variables as mean ± SD and median (min–max). Normality was assessed with the Kolmogorov–Smirnov test. Group comparisons were made with the *t*-test or Mann–Whitney U test; categorical variables were compared with the chi-square or Fisher’s exact test. Correlations were examined with the Spearman coefficient (r; 0.10–0.29 weak, 0.30–0.69 moderate, ≥0.70 strong). Multivariable binary logistic regression analysis was performed to identify factors independently associated with surgical success. Because of the limited number of events (21 unsuccessful cases), and in order to avoid overfitting, only three variables carrying clinically distinct information were entered into the model from among the ultrasonographic parameters found to be significant in univariate analysis (SP-TAPE distance during Valsalva, concordant sling–urethral movement, and bladder wall thickness). Questionnaire scores, which were measured concurrently with surgical success, were not included in the model because they overlap with the outcome itself. Variables were entered simultaneously (Enter method); model fit was assessed with the Hosmer–Lemeshow test, and results are reported as ORs with 95% CIs. There were no missing data for any of the variables analysed; all analyses were therefore based on the complete set of 74 patients, except for dyspareunia, which was assessed in the 61 sexually active patients. Significance was accepted at *p* < 0.05.

## 3. Results

### 3.1. Participants

A total of 74 female patients were included in the analysis. Demographic and clinical characteristics are presented in Table 1. Mean age was 49.1 ± 6.5 years, mean BMI was 29.5 ± 5.1 kg/m^2^, and mean time since surgery was 43.7 ± 18.4 months. Of the patients, 50.0% were postmenopausal, and 82.4% were sexually active.

### 3.2. Postoperative Outcomes

The distribution of postoperative symptoms is shown in Table 2. Surgical success was achieved in 53 patients (71.6%); persistent stress incontinence was present in 21 patients (28.4%). Urge incontinence was reported in 41.9%, mixed incontinence in 17.6%, and pelvic pain in 31.1%. Dyspareunia was present in 30 of the 61 sexually active patients (49.2%). Overall, 68 patients (91.9%) reported satisfaction with the outcome of surgery. A response of “very satisfied” on the Likert scale was given by 73.6% in the successful group and 9.5% in the unsuccessful group. Mesh extrusion was identified in one patient (1.4%); this patient was not sexually active and was therefore not included in the dyspareunia analysis. No other case of mesh exposure or extrusion was detected.

Mean questionnaire scores were as follows: I-QOL 84.80 ± 15.89; UDI-6 3.70 ± 2.78; IIQ-7 2.89 ± 4.43; OAB-V8 8.89 ± 7.89.

### 3.3. Ultrasonographic Findings

Transperineal ultrasound measurements are summarised in Table 3. The sling was most frequently located at the mid-urethral level. At rest, the sling was located at the mid-urethra in 50 patients (67.6%), at the distal urethra in 14 patients (18.9%) and at the proximal urethra in 10 patients (13.5%); during Valsalva, it was located at the mid-urethra in 44 patients (59.5%), at the proximal urethra in 20 patients (27.0%) and at the distal urethra in 10 patients (13.5%). In the sagittal plane, sling shape at rest was straight in 48 cases (64.9%), C-shaped in 24 cases (32.4%), and S-shaped in 2 cases (2.7%); during Valsalva, it was straight in 47 cases (63.5%), C-shaped in 24 cases (32.4%), and S-shaped in 3 cases (4.1%). Concordant movement of the sling with the urethra was observed in 52 patients (70.3%). Sling deformation was detected in 21 patients (28.4%), and a curved sling arm morphology was present in 50.0% of patients in the axial plane.

### 3.4. Factors Associated with Surgical Success

No differences were found between the successful and unsuccessful groups with respect to demographic and clinical variables (all *p* > 0.05). In contrast, I-QOL scores were significantly higher, and UDI-6, IIQ-7, and OAB-V8 scores were significantly lower in the successful group (all *p* < 0.001).

The comparison of ultrasonographic parameters is presented in Table 4. Surgical success was associated with mid-urethral sling location (rest: 77.4% vs. 42.9%, *p* = 0.011; Valsalva: 71.7% vs. 28.6%, *p* = 0.002), a shorter SP-TAPE distance (rest: 17.32 ± 2.12 vs. 20.24 ± 3.70 mm, *p* = 0.004; Valsalva: 12.13 ± 1.91 vs. 15.24 ± 2.27 mm, *p* < 0.001), concordant sling–urethral movement (81.1% vs. 42.9%, *p* = 0.001), and a lower bladder wall thickness (2.89 ± 1.12 vs. 3.75 ± 1.24 mm, *p* = 0.009). Sling arm morphology (*p* = 0.797), sling deformation (*p* = 0.243), BND, and PVR were not associated with success (all *p* > 0.05).

### 3.5. Correlations

Correlations between questionnaire scores and ultrasonographic measurements are presented in Table 5. Bladder wall thickness showed moderate significant correlations with all four instruments: I-QOL (r = −0.604), UDI-6 (r = 0.576), IIQ-7 (r = 0.551), and OAB-V8 (r = 0.667) (all *p* < 0.001). Weak correlations were found between SP-TAPE during Valsalva and I-QOL (r = −0.263, *p* = 0.024) and UDI-6 (r = 0.274, *p* = 0.018).

### 3.6. Sling Arm Morphology and Postoperative Dyspareunia

The relationship between axial sling arm morphology and postoperative symptoms is shown in Table 6. Dyspareunia was assessed only in sexually active patients (*n* = 61) and was significantly more frequent in patients with curved arms than in those with non-curved arms (75.0% vs. 27.3%; *p* < 0.001; OR = 8.00, 95% CI: 2.54–25.22). Of the 13 sexually inactive patients, 9 had curved and 4 had non-curved arm morphology; the distribution of these patients did not differ significantly between the groups (*p* = 0.222). No differences were found between the groups with respect to pelvic pain or urge incontinence, which were assessed in the whole cohort (*p* = 0.079 and *p* = 0.814, respectively). Among sexually active patients, the rest–Valsalva BND difference was numerically higher in those with dyspareunia, but the difference did not reach statistical significance (17.10 ± 7.52 vs. 14.03 ± 8.25 mm; *p* = 0.134).

### 3.7. Factors Independently Associated with Surgical Success

The multivariable logistic regression analysis is presented in Table 7. Three variables were independently associated with surgical success: SP-TAPE distance during Valsalva (OR = 0.337; 95% CI: 0.181–0.629; *p* = 0.001), concordant sling–urethral movement (OR = 8.963; 95% CI: 1.612–49.857; *p* = 0.012), and bladder wall thickness (OR = 0.375; 95% CI: 0.185–0.759; *p* = 0.006). The model was significant overall (likelihood ratio test *p* < 0.001), and the Hosmer–Lemeshow goodness-of-fit test indicated acceptable calibration (*p* = 0.177); the correct classification rate of the model was 85.1%.

## 4. Discussion

### 4.1. Overview

Although midurethral sling surgery is one of the gold-standard treatments for stress urinary incontinence, the mechanisms determining surgical success and the development of postoperative complications remain incompletely understood. Objective assessment of postoperative anatomical and functional changes is therefore of considerable importance both for the accurate interpretation of surgical outcomes and for the optimisation of patient management. In this study, the static and dynamic characteristics of the sling assessed by transperineal ultrasound were examined comprehensively, together with surgical success and postoperative complications. Our findings point to three observations. First, surgical success appeared to be associated with appropriate anatomical positioning of the sling, its concordant movement with the urethra, and its dynamic proximity to the symphysis pubis during the Valsalva manoeuvre. Second, although sling arm morphology had no appreciable effect on surgical success, it appeared to be associated with postoperative dyspareunia. Finally, the significant correlation between bladder wall thickness and patient-reported symptom severity suggests that this measurement may serve as a complementary ultrasonographic marker in postoperative assessment.

### 4.2. Surgical Success and Patient-Reported Outcomes

In our study, the objective surgical success rate was 71.6%, and the subjective satisfaction rate was 91.9%. These results are largely consistent with previous studies reporting the efficacy of TOT surgery. In the meta-analysis by Maggiore et al. (≥5 years of follow-up), cumulative objective and subjective cure rates for TOT were reported as 76.5% and 81.3%, respectively [24], whereas in the Cochrane review by Ford et al., subjective improvement rates ranged from 62% to 98% in the primary comparison, which comprised 36 trials and 5514 women [25]. The consistency of our findings with these large-scale studies supports the reliability and external validity of our results.

One notable finding of our study was that the subjective satisfaction rate was markedly higher than the objective surgical success rate. This divergence suggests that the success of TOT surgery may not be explained by the restoration of continence alone and that a reduction in symptom burden, improvement in quality of life, and fulfilment of patients’ treatment expectations may also contribute to satisfaction. Indeed, in recent years the evaluation of incontinence surgery has increasingly emphasised patient-reported outcome measures in addition to objective cure rates. In this context, the finding that all four validated quality-of-life and symptom instruments (I-QOL, UDI-6, IIQ-7 and OAB-V8) yielded significantly better scores in the successful group (all *p* < 0.001) and that a response of “very satisfied” on the Likert scale was reported by 73.6% in the successful group compared with 9.5% in the unsuccessful group indicates that a degree of agreement between objective surgical outcomes and patient-perceived improvement is nevertheless preserved despite generally high satisfaction levels. These findings suggest that it may be useful to consider patient-centred outcome measures alongside objective continence rates in the evaluation of TOT surgery.

### 4.3. Sling Location and Concordant Movement

Assessments performed with transperineal ultrasound suggested that midurethral sling location and dynamic sling–urethral concordance may be decisive for surgical success. The finding that the sling was most frequently positioned at the mid-urethral level at rest (67.6%) was consistent with the literature emphasising the central role of this segment in the continence mechanism [12,26]. The decrease in the proportion of mid-segment positioning and the marked increase in proximal positioning during the Valsalva manoeuvre (27.0%) suggest that the sling may be displaced under dynamic stress and that this may affect functional outcomes.

The markedly higher proportion of mid-urethral positioning in the successful group both at rest (77.4%) and during Valsalva (71.7%) suggests that preservation of mid-segment support under static and dynamic conditions may be important for the continence mechanism [27]. Conversely, the markedly lower proportion of mid-urethral positioning in the unsuccessful group appears consistent with the concept of a “functionally inadequate sling position” described in the literature [28]. One notable finding of our study was that concordant movement of the sling with the urethra emerged as one of the factors independently associated with surgical success in multivariable analysis. The concordant movement rate reached 81.1% in the successful group, whereas it remained at 42.9% in the unsuccessful group, and concordant movement emerged as an independently associated factor in multivariable analysis (OR = 8.963; 95% CI: 1.612–49.857; *p* = 0.012), suggesting that sling function may not be limited to the provision of anatomical support alone. Our findings are broadly consistent with the study by Hegde et al., who reported concordant movement in all successful cases but in only one-third of unsuccessful cases [14]. Taken together, these results suggest that the midurethral sling may behave less as a passive support material than as a dynamic support system that moves physiologically with the urethra during increases in intra-abdominal pressure.

In contrast, no significant relationship was found between sling deformation and surgical success. This finding differs in part from the results of Hegde et al. [14]. This difference may arise from the location and degree of deformation and its interaction with urethral movement rather than from the presence of deformation itself. Differences in the sling material used, surgical technique, patient population, and assessment methods are also among the possible factors that could explain the discrepancy between studies. For this reason, we consider that sling deformation may be more appropriately interpreted together with other dynamic ultrasonographic parameters rather than being evaluated as a criterion of success on its own. Our findings suggest that it may be useful to consider not only sling location but also sling–urethral movement concordance in postoperative transperineal ultrasound assessment. Such an approach may help in understanding the mechanisms of failure and, if confirmed, could inform postoperative assessment; however, because all measurements in the present study were obtained at a single visit, this proposal requires confirmation in prospective studies with larger samples before it can be translated into clinical practice. Such an approach may help in understanding the mechanisms of failure and, if confirmed, could inform postoperative assessment; however, because all measurements in the present study were obtained at a single visit, this proposal requires confirmation in prospective studies with larger samples before it can be translated into clinical practice.

### 4.4. SP-TAPE Distance and Dynamic Functional Assessment

In our study, the symphysis pubis–sling distance (SP-TAPE) was shown to be closely associated with surgical success, particularly during the Valsalva manoeuvre. Although the SP-TAPE distance was significantly lower in successful cases both at rest and during Valsalva, only the value measured during Valsalva was independently associated with surgical success in multivariable logistic regression analysis (OR = 0.337; 95% CI: 0.181–0.629; *p* = 0.001). This finding suggests that the functional behaviour of the sling under dynamic loading is more decisive for surgical outcomes than its static anatomical position.

Whereas measurements obtained at rest reflect the anatomical position of the sling, those obtained during the Valsalva manoeuvre provide a more realistic representation of the functional support that the sling provides to the urethra under increased intra-abdominal pressure. An increased SP-TAPE distance during Valsalva therefore suggests that the sling fails to provide adequate and stable support to the urethra under stress conditions and that the effectiveness of the continence mechanism is consequently reduced. Our findings indicate that dynamic ultrasonographic assessment may have greater clinical value than static measurements in predicting functional success.

Our results are consistent with the study by Pędraszewski et al., who reported a shorter tape–symphysis distance in successful cases [29]. Our study additionally shows that this relationship cannot be explained by anatomical proximity alone and that dynamic assessment contributes to a more comprehensive interpretation of surgical success by providing Supplementary Information. In other words, what matters for surgical success is not only that the sling is positioned close to the pubic symphysis but also that this relationship is preserved during increases in intra-abdominal pressure. This finding suggests that SP-TAPE measurement, particularly together with values obtained during the Valsalva manoeuvre, is a useful ultrasonographic parameter that may be taken into account when interpreting surgical outcome in postoperative transperineal ultrasound assessment. Including dynamic SP-TAPE measurement, and not only sling location, in postoperative TPUS examinations may therefore contribute to the early identification of patients at risk of surgical failure and to the individualisation of patient management.

### 4.5. Arm Morphology: The Distinction Between Surgical Success and Dyspareunia

One of the original findings of our study was the observation that sling arm morphology displayed two distinct and mutually independent patterns of association with surgical success and with postoperative dyspareunia. Although a curved arm morphology was detected in half of the patients (50.0%) in the axial plane, arm morphology did not differ between the successful and unsuccessful groups (*p* = 0.797); similarly, sling deformation was not associated with surgical success (*p* = 0.243). These findings indicate that the morphological characteristics of the sling arms may not play a decisive role in the continence mechanism.

These results appear broadly consistent with the findings of Spelzini et al. [30]. In that study, the type of anchorage defined by whether the sling arms crossed the obturator membrane (bilateral, unilateral, or no anchorage) was reported not to affect surgical success; although the arms did not cross the obturator membrane bilaterally in 77% of patients, this was reported not to have an adverse effect on surgical efficacy. The authors emphasised that the determinant of surgical success may be the mid-urethral position and functional effect of the sling rather than the site of anchorage. On the other hand, Illiano et al. reported arm asymmetry as an independent risk factor for surgical failure (OR = 4.47; *p* < 0.0001) [8]. The divergence of our findings from those of Illiano et al. may arise from methodological differences in the type of sling used, the patient population, and the way asymmetry was defined.

Another clinically notable finding of our study was the significant association between sling arm morphology and postoperative dyspareunia. Among sexually active patients, the rate of dyspareunia was 75.0% in those with curved arms, whereas it remained at 27.3% in those with non-curved arms (*p* < 0.001; OR = 8.00, 95% CI: 2.54–25.22). That arm morphology was not associated with surgical success but showed a significant association with dyspareunia suggests that this parameter may be of clinical importance through mechanical interaction with the surrounding tissues rather than through the continence mechanism. To our knowledge, this relationship has not previously been addressed systematically in the literature. Given the cross-sectional design, the sample size, and the absence of preoperative information on dyspareunia, we regard this association as an exploratory finding rather than as evidence of a causal relationship, and we present it as a hypothesis for prospective testing.

Bladder neck mobility was numerically higher in patients with dyspareunia, but this difference did not reach significance (Section 3.6); this mechanism could therefore not be confirmed with our data. It may be postulated that curving of the arms creates heterogeneous tension on the vaginal and paraurethral tissues and that accompanying increased bladder neck mobility may contribute to dyspareunia through mechanical friction, local rigidity, and functional mismatch with the surrounding tissue during intercourse [31]. Further studies are nevertheless required to confirm this mechanism.

Clinically, these findings point to the potential value of a surgical approach that addresses two distinct objectives simultaneously. Correct anatomical positioning of the sling, its concordant movement with the urethra, and its dynamic tension may contribute to the restoration of continence, whereas straight, non-curved placement of the arms might contribute to the prevention of postoperative pain and sexual dysfunction. We emphasise that the latter is a hypothesis generated by the present observations and not a conclusion supported by them; it would require testing in a prospective study with preoperative and postoperative assessment of sexual function. These findings suggest that postoperative quality of life and sexual function, and not only continence rates, may be important considerations in the evaluation of midurethral sling surgery.

### 4.6. Bladder Wall Thickness and Symptom Burden

Another notable finding of our study is that bladder wall thickness (BWT) showed a consistent and moderate correlation with patient-reported symptom burden. BWT showed moderately significant correlations with all four validated questionnaires: I-QOL (r = −0.604), UDI-6 (r = 0.576), IIQ-7 (r = 0.551), and OAB-V8 (r = 0.667) (all *p* < 0.001). BWT was also significantly lower in the successful group than in the unsuccessful group (2.89 ± 1.12 vs. 3.75 ± 1.24 mm; *p* = 0.009). Indeed, BWT was also independently associated with surgical success in multivariable analysis (OR = 0.375; 95% CI: 0.185–0.759; *p* = 0.006). These findings suggest that increased detrusor wall thickness may be associated with both symptom severity and surgical failure. Given that detrusor hypertrophy is associated in the literature with chronic obstruction and overactive bladder, BWT emerges as a useful ultrasonographic parameter in the assessment of postoperative symptom burden. That this specific correlation has not previously been examined systematically in the literature adds particular value to our finding. Because bladder wall thickness varies with bladder filling, we repeated the correlation analysis adjusting for post-void residual volume; the coefficients were essentially unchanged (I-QOL r = −0.596; UDI-6 r = 0.570; IIQ-7 r = 0.543; OAB-V8 r = 0.661; all *p* < 0.001), indicating that the association is not explained by differences in bladder filling. No preoperative measurement was available for comparison, however, and this association therefore also requires confirmation in prospective studies with standardised filling conditions. 

### 4.7. Strengths and Limitations

The principal strength of our study is the independent and blinded assessor approach applied in outcome assessment. Patient-reported outcomes and physical examination were assessed by an independent physician not involved in the surgical process, whereas the transperineal ultrasound examination was performed by a second investigator blinded to the clinical symptoms, questionnaire results, and physical examination findings. This approach minimised assessor bias and allowed a more objective evaluation of the relationship between ultrasonographic findings and clinical outcomes. In addition, the combined use of four validated patient-reported outcome measures (I-QOL, UDI-6, IIQ-7, and OAB-V8) permitted a multidimensional assessment of postoperative symptom burden and quality of life. Furthermore, because patients who developed postoperative overactive bladder and obstructive voiding were not excluded from the study, the ultrasonographic characteristics of these complications could also be examined comprehensively.

The study nevertheless has certain limitations. First, the cross-sectional design, in which patients were identified retrospectively, does not entirely eliminate the risk of selection and information bias inherent in observational studies. Because data on preoperative symptom scores and preoperative dyspareunia status were not available, it cannot be determined whether postoperative dyspareunia was de novo or persistent; the relationship between sling arm morphology and dyspareunia should therefore be interpreted with caution. The dyspareunia analysis was restricted to sexually active patients only; because cessation of sexual activity may itself be a consequence of dyspareunia, this approach is conservative and may underestimate the true effect. In addition, because the sample size was limited to the available archival records, no a priori power analysis was performed, and no correction for multiple comparisons was applied; weak correlations should therefore be regarded as hypothesis-generating findings. There is also a limitation with respect to reachability: In total, 53 of the 148 patients initially identified could not be contacted for the follow-up examination. However, the great majority of these patients were found either to have died or to have migrated to other cities following the Kahramanmaraş earthquakes of 6 February 2023, which directly affected the study region (Hatay). This suggests that the loss to follow-up was attributable to an external and random event unrelated to surgical outcome or incontinence severity and minimises the risk of selection bias in the conventional sense; nevertheless, the fact that the study population consisted of patients who were reachable in the post-earthquake period should be taken into account when generalising the results. Concomitant vaginal repair was performed in 12 patients (16.2%). Because such procedures may independently contribute to postoperative pain and sexual discomfort, they represent a potential confounder for the association between sling arm morphology and dyspareunia; the available records did not permit a reliable separate analysis of this subgroup, and this association should therefore be interpreted with the possibility of residual confounding in mind. Intraobserver and interobserver reliability of the ultrasonographic measurements were not formally assessed, and the classification of sling arm morphology in particular involves a degree of subjective interpretation; our findings should be confirmed in studies in which reproducibility is documented. The interval between surgery and assessment was wide, ranging from 12 to 76 months, and both symptoms and ultrasonographic appearances may change over this period; because each patient was examined only once, we could not determine whether the associations we report vary with time since surgery. Bladder wall thickness was measured at a mean bladder volume of 82.1 mL, which is above the threshold of 50 mL usually recommended for detrusor wall thickness measurement, so our absolute values are not directly comparable with published thresholds. Bladder volume during the cough stress test was defined only as full filling and was not standardised, which may have affected the sensitivity of the test. The postoperative onset of urge incontinence was established from patient report rather than from documented preoperative assessment and is therefore subject to recall bias. All operations were performed by a single surgeon, which removes inter-surgeon variability but limits the generalisability of the findings to surgeons of differing experience. The fact that the study was conducted at a single centre is a further element that may limit the generalisability of the findings to different patient populations. Finally, because of the study design, the observed relationships indicate possible associations rather than establishing causality. It is therefore important that our findings be confirmed in larger, multicentre and prospective studies, and in particular that the effects of sling arm morphology on postoperative pain, dyspareunia, and sexual function be investigated using standardised ultrasonographic protocols.

## 5. Conclusions

This study indicates that success in midurethral sling surgery should not be assessed solely by the restoration of continence and that considering the anatomical location of the sling, its dynamic concordance with the urethra and patient-reported outcomes together may provide a more comprehensive assessment. Dynamic parameters assessed by transperineal ultrasound, particularly the symphysis pubis–sling distance measured during Valsalva and concordant sling–urethral movement, showed a strong relationship with surgical success and emerged as important ultrasonographic markers associated with it. In contrast, the finding that sling arm morphology was not associated with surgical success but showed a significant association with postoperative dyspareunia suggests that continence and patient quality of life are outcome measures that may be assessed independently of one another. In the absence of preoperative data on dyspareunia, the latter association should be regarded as exploratory and requires confirmation in prospective studies.

These findings indicate that postoperative transperineal ultrasound may be a useful tool not only for anatomical assessment but also for the holistic evaluation of surgical efficacy and complication risk. The results also demonstrate the importance of taking postoperative pain, sexual function, and patient quality of life into account, and not continence alone, in surgical techniques and sling designs to be developed in the future. All findings relate to the outside-in transobturator sling, the only type evaluated in this study, and should not be extrapolated to other midurethral sling techniques. Future studies should also examine how surgical success is modified by the underlying pattern of pelvic floor dysfunction, particularly in women in whom SUI and POP coexist—a group deliberately excluded from the present study, since POP-Q stage 2 or above was an exclusion criterion, in order to isolate the anatomical determinants of sling function. Confirmation of our findings in larger, multicentre and prospective studies will help to define more clearly the place of these ultrasonographic parameters in clinical practice.

## Figures and Tables

**Figure 1:**
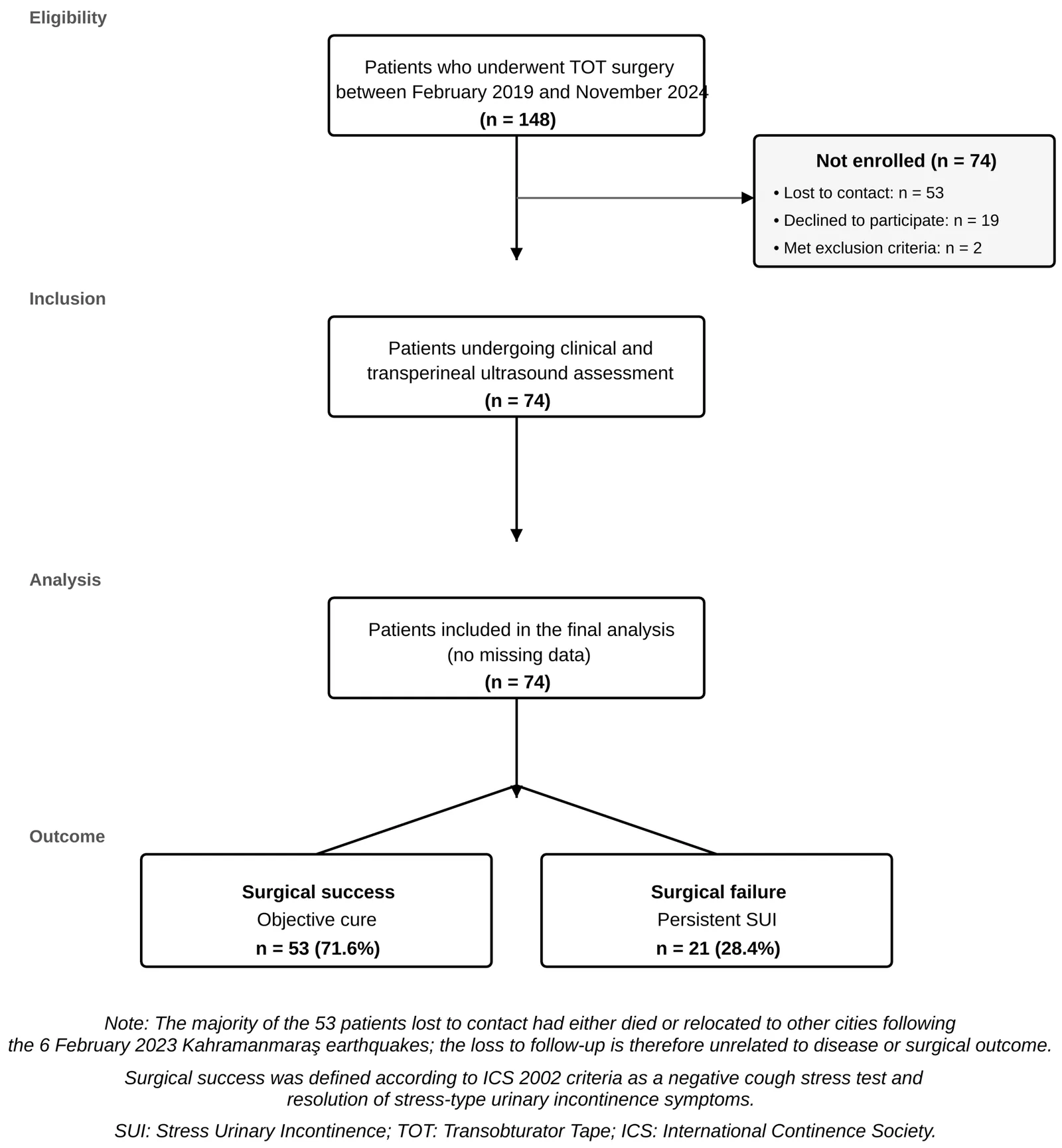
Patient flow diagram.

**Figure 2:**
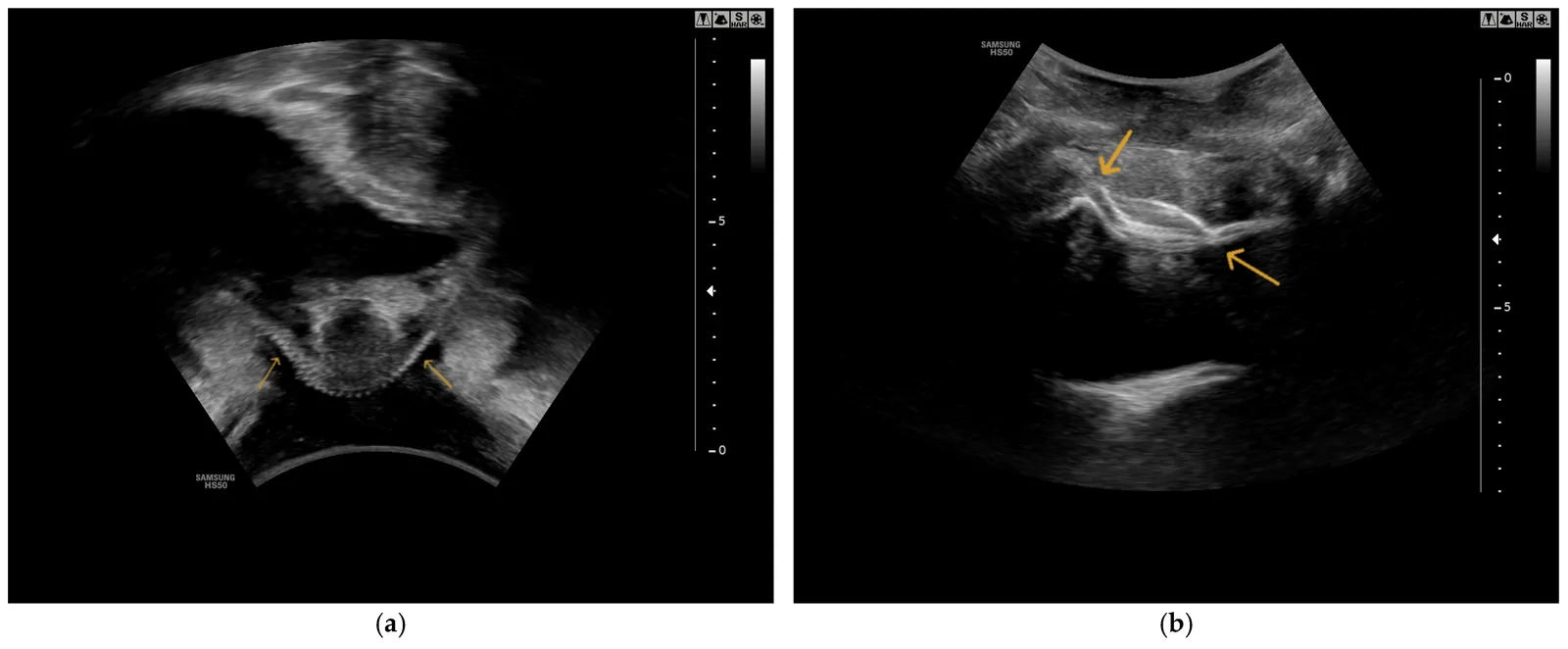
Sling arm morphology assessed by transperineal ultrasound in the axial plane: (**a**) Non-curved sling arms—both arms follow a straight course. (**b**) Curved sling arms—one or both arms deviate from a straight course. Arrows indicate the sling arms.

**Table 1:** Demographic and clinical characteristics of the patients (*n* = 74).

Variable		Mean ± SD	Median (Min–Max)
Age (years)		49.08 ± 6.48	50 (33–63)
Gravidity		4.26 ± 1.87	4 (0–12)
Parity		3.50 ± 1.17	4 (0–6)
Time since surgery (months)		43.67 ± 18.36	41 (12–76)
BMI (kg/m^2^)		29.46 ± 5.07	28.6 (16.9–41.0)
Menopause	Present, *n* (%)	37 (50.0%)	—
	Absent, *n* (%)	37 (50.0%)	
Sexual activity	Present, *n* (%)	61 (82.4%)	—
	Absent, *n* (%)	13 (17.6%)	
Smoking	Present, *n* (%)	18 (24.3%)	—
	Absent, *n* (%)	56 (75.7%)	
Alcohol	Present, *n* (%)	15 (20.3%)	—
	Absent, *n* (%)	59 (79.7%)	

SD: standard deviation; BMI: body mass index.

**Table 2:** Postoperative incontinence and pain status (*n* = 74).

Variable	*n* (%)
Stress incontinence	21 (28.4%)
Urge incontinence	31 (41.9%)
Mixed incontinence	13 (17.6%)
Pelvic pain	23 (31.1%)
Dyspareunia	30/61 (49.2%) a

a—Dyspareunia was assessed only in sexually active patients (*n* = 61). All other variables were assessed in the whole cohort (*n* = 74).

**Table 3:** Transperineal ultrasound measurements (*n* = 74).

Variable	Mean ± SD	Median (Min–Max)
SP-TAPE at rest (mm)	18.15 ± 2.95	17.5 (11–29)
SP-TAPE during Valsalva (mm)	13.01 ± 2.45	13 (8–20)
BND at rest (mm)	21.88 ± 3.23	21 (15–31)
BND during Valsalva (mm)	6.73 ± 7.67	7 (−10–21)
BND rest–Valsalva difference (mm)	15.15 ± 7.80	15 (0–31)
PVR (mL)	82.09 ± 20.68	80 (45–136)
BWT (mm)	3.14 ± 1.21	3 (1–6)
Sling location at rest—mid-urethra, *n* (%)	50 (67.6%)	—
Sling location at rest—distal urethra, *n* (%)	14 (18.9%)	—
Sling location at rest—proximal urethra, *n* (%)	10 (13.5%)	—
Sling location during Valsalva—mid-urethra, *n* (%)	44 (59.5%)	—
Sling location during Valsalva—proximal urethra, *n* (%)	20 (27.0%)	—
Sling location during Valsalva—distal urethra, *n* (%)	10 (13.5%)	—
Sling shape at rest—straight, *n* (%)	48 (64.9%)	—
Sling shape at rest—C, *n* (%)	24 (32.4%)	—
Sling shape at rest—S, *n* (%)	2 (2.7%)	—
Sling shape during Valsalva—straight, *n* (%)	47 (63.5%)	—
Sling shape during Valsalva—C, *n* (%)	24 (32.4%)	—
Sling shape during Valsalva—S, *n* (%)	3 (4.1%)	—
Concordant movement—present, *n* (%)	52 (70.3%)	—
Deformation—present, *n* (%)	21 (28.4%)	—
Axial—curved arms, *n* (%)	37 (50.0%)	—

SP-TAPE: symphysis pubis–sling distance; BND: bladder neck descent; PVR: post-void residual; BWT: bladder wall thickness. Sling shape was classified into four categories (straight, S, C, and other); the “other” category was not observed in any case.

**Table 4:** Comparison of ultrasonographic measurements according to surgical success (*n* = 74).

Variable	Unsuccessful (*n* = 21)	Successful (*n* = 53)	*p*
Mid-urethra—at rest, *n* (%)	9 (42.9%)	**41 (77.4%)**	**0.011**
Mid-urethra—during Valsalva, *n* (%)	6 (28.6%)	**38 (71.7%)**	**0.002**
SP-TAPE at rest (mm)	20.24 ± 3.70	17.32 ± 2.12	**0.004**
SP-TAPE during Valsalva (mm)	15.24 ± 2.27	12.13 ± 1.91	**<0.001**
Concordant movement—present, *n* (%)	9 (42.9%)	**43 (81.1%)**	**0.001**
BWT (mm)	3.75 ± 1.24	2.89 ± 1.12	**0.009**
BND at rest (mm)	21.86 ± 2.78	21.89 ± 3.41	0.827
PVR (mL)	83.67 ± 21.25	81.47 ± 20.62	0.833
Deformation—present, *n* (%)	8 (38.1%)	13 (24.5%)	0.243
Curved arms, *n* (%)	11 (52.4%)	26 (49.1%)	0.797

Mann–Whitney U/*t*-test; Pearson’s chi-square. Values in bold are statistically significant.

**Table 5:** Spearman correlations between questionnaire scores and ultrasonographic measurements (*n* = 74).

Measurement	I-QOL	UDI-6	IIQ-7	OAB-V8
SP-TAPE during Valsalva (mm)	−0.263 *	0.274 *	0.156	0.109
BND difference (mm)	−0.049	0.194	0.022	0.066
PVR (mL)	−0.118	0.105	0.109	0.124
BWT (mm)	**−0.604 †**	**0.576 †**	**0.551 †**	**0.667 †**

Spearman correlation coefficient (r). * Values in bold are statistically significant. *p* < 0.05; † *p* < 0.001.

**Table 6:** Comparison of postoperative dyspareunia, pain, and urge symptoms according to axial sling arm morphology.

Symptom	Non-Curved Arms	Curved Arms	*p*
Dyspareunia—present, *n* (%) a	9/33 (27.3%)	**21/28 (75.0%)**	**<0.001**
Pelvic pain—present, *n* (%)	8/37 (21.6%)	15/37 (40.5%)	0.079
Urge incontinence—present, *n* (%)	15/37 (40.5%)	16/37 (43.2%)	0.814

a—Dyspareunia was assessed only in sexually active patients (*n* = 61; non-curved arms *n* = 33, curved arms *n* = 28). Thirteen sexually inactive patients (9 with curved and 4 with non-curved arms) were excluded from this analysis. Pelvic pain and urge incontinence were assessed in the whole cohort (*n* = 74). Pearson’s chi-square test. Values in bold are statistically significant.

**Table 7:** Factors independently associated with surgical success: multivariable logistic regression analysis.

Variable	OR	95% CI	*p*
SP-TAPE during Valsalva (mm)	**0.337**	**0.181–0.629**	**0.001**
BWT (mm)	0.375	0.185–0.759	0.006
Concordant movement—present	**8.963**	**1.612–49.857**	**0.012**

OR: odds ratio; CI: confidence interval; BWT: bladder wall thickness. Dependent variable: objective surgical success (1 = successful). Variables were entered simultaneously (Enter method). Model: likelihood ratio test *p* < 0.001; Hosmer–Lemeshow *p* = 0.177; correct classification rate 85.1%. Values in bold are statistically significant.

## Data Availability

The data supporting the findings of this study are available from the corresponding author upon reasonable request. The data are not publicly available because of patient confidentiality.

## Supplementary Materials

The following supporting information can be downloaded at: https://www.mdpi.com/article/10.3390/jcm15197613/s1. Table S1: STROBE checklist.

## Abbreviations

BMI: body mass index; BND, bladder neck descent; BWT, bladder wall thickness; CI, confidence interval; ICS, International Continence Society; IIQ-7, Incontinence Impact Questionnaire—Short Form; I-QOL, Incontinence Quality of Life; OAB, overactive bladder; OAB-V8, Overactive Bladder Awareness Tool; OR, odds ratio; POP, pelvic organ prolapse; PVR, post-void residual; SD, standard deviation; SP-TAPE, symphysis pubis–sling distance; SUI, stress urinary incontinence; TOT, transobturator tape; TPUS, transperineal ultrasound; UDI-6, Urogenital Distress Inventory—Short Form.
