# Supplementary material for: Assessment of Sling Characteristics After Transobturator Tape Placement Using Transperineal Ultrasonography: Relationship with Continence and Dyspareunia—A Cross-Sectional Study
Source: J Clin Med. 2026 Oct 1;15(19):7613. doi: 10.3390/jcm15197613 (PMC13665933; doi:10.3390/jcm15197613)
Supplement: Supplementary file 1 [file jcm-15-07613-s001.zip › jcm-4502405-supplementary.pdf]

STROBE Statement—Checklist of items that should be included in reports of cross-sectional studies

Manuscript jcm-4502405 (revised version): "Assessment of Sling Characteristics After Transobturator Tape Placement Using Transperineal Ultrasonography: Relationship with Continence and Dyspareunia — A Cross-Sectional Study"

	Item No	Recommendation	Section and page no.
**Title and abstract**	1	(a) Indicate the study's design with a commonly used term in the title or the abstract	Title ("— A Cross-Sectional Study"), p. 1; Abstract, Methods, p. 1
		(b) Provide in the abstract an informative and balanced summary of what was done and what was found	Abstract, pp. 1–2
**INTRODUCTION**			
**Background / rationale**	2	Explain the scientific background and rationale for the investigation being reported	1. Introduction, pp. 2–3
**Objectives**	3	State specific objectives, including any prespecified hypotheses	1. Introduction (final paragraph), p. 3; primary endpoint, 2.1 Study Design and Ethical Approval, p. 3
**METHODS**			
**Study design**	4	Present key elements of study design early in the paper	2.1 Study Design and Ethical Approval, p. 3
**Setting**	5	Describe the setting, locations, and relevant dates, including periods of recruitment, exposure, follow-up, and data collection	2.1 Study Design and Ethical Approval, p. 3
**Participants**	6	(a) Give the eligibility criteria, and the sources and methods of selection of participants	2.2 Participants and Sample, pp. 4–5
**Variables**	7	Clearly define all outcomes, exposures, predictors, potential confounders, and effect modifiers. Give diagnostic criteria, if applicable	2.3 Clinical Assessment and Instruments, p. 6; 2.5 Ultrasonographic Parameters, p. 7; potential confounders (concomitant vaginal repair, single surgeon), 2.2, p. 4 and 4.7 Strengths and Limitations, p. 22
**Data sources / measurement**	8*	For each variable of interest, give sources of data and details of methods of assessment (measurement). Describe comparability of assessment methods if there is more than one group	2.3 Clinical Assessment and Instruments, p. 6; 2.4 Physical Examination and Ultrasonography, p. 6 (examination sequence, bladder filling conditions, equipment and assessor experience); 2.5 Ultrasonographic Parameters, p. 7
**Bias**	9	Describe any efforts to address potential sources of bias	2.4 Physical Examination and Ultrasonography (independent, blinded assessors), p. 6; 4.7 Strengths and Limitations, pp. 21–22
**Study size**	10	Explain how the study size was arrived at	2.2 Participants and Sample, p. 5; 4.7 Strengths and Limitations, p. 22
**Quantitative variables**	11	Explain how quantitative variables were handled in the analyses. If applicable, describe which groupings were chosen and why	2.6 Statistical Analysis, p. 8; groupings: 2.5 Ultrasonographic Parameters (urethral thirds; curvature as the sole morphological criterion), p. 7; 2.3 Clinical Assessment and Instruments (Likert grouping), p. 6
**Statistical methods**	12	(a) Describe all statistical methods, including those used to control for confounding	2.6 Statistical Analysis, p. 8
		(b) Describe any methods used to examine subgroups and interactions	3.6 Sling Arm Morphology and Postoperative Dyspareunia, p. 14; Table 6 footnote, p. 14
		(c) Explain how missing data were addressed	2.6 Statistical Analysis, p. 8 (no missing data for any variable analysed); Figure 1, p. 5
		(d) If applicable, describe analytical methods taking account of sampling strategy	Not applicable — no probability sampling; all eligible patients within the archival period were identified and invited consecutively (2.2 Participants and Sample, pp. 4–5)
		(e) Describe any sensitivity analyses	No formal sensitivity analysis was performed; the dyspareunia analysis was deliberately restricted to sexually active patients as a conservative approach (4.7 Strengths and Limitations, p. 21)
**RESULTS**			
**Participants**	13*	(a) Report numbers of individuals at each stage of study—eg numbers potentially eligible, examined for eligibility, confirmed eligible, included in the study, completing follow-up, and analysed	2.2 Participants and Sample, p. 5; Figure 1, p. 5; 3.1 Participants, p. 8
		(b) Give reasons for non-participation at each stage	2.2 Participants and Sample, p. 5; 4.7 Strengths and Limitations, p. 22
		(c) Consider use of a flow diagram	Figure 1 (patient flow diagram), p. 5
**Descriptive data**	14*	(a) Give characteristics of study participants (eg	3.1 Participants, p. 8; Table 1, p. 9; 3.3 Ultrasonographic

		demographic, clinical, social) and information on exposures and potential confounders	Findings, p. 10; Table 3, p. 11
		(b) Indicate number of participants with missing data for each variable of interest	No missing data (2.6 Statistical Analysis, p. 8); dyspareunia denominators given in the footnotes to Table 2, p. 10, and Table 6, p. 14
**Outcome data**	15*	Report numbers of outcome events or summary measures	3.2 Postoperative Outcomes, pp. 9–10 (including mesh extrusion); Table 2, p. 10
**Main results**	16	(a) Give unadjusted estimates and, if applicable, confounder-adjusted estimates and their precision (eg, 95% confidence interval). Make clear which confounders were adjusted for and why they were included	Unadjusted: 3.4 Factors Associated with Surgical Success, p. 12; Table 4, p. 12. Adjusted with 95% CI: 3.7 Factors Independently Associated with Surgical Success, p. 15; Table 7, p. 15. Variable-selection rationale: 2.6 Statistical Analysis, p. 8
		(b) Report category boundaries when continuous variables were categorized	2.5 Ultrasonographic Parameters (urethra divided into three equal segments), p. 7; 2.6 Statistical Analysis (correlation strength bands), p. 8
		(c) If relevant, consider translating estimates of relative risk into absolute risk for a meaningful time period	Not applicable — cross-sectional design; no incidence or time-based risk estimates were derived. Absolute proportions are reported alongside all odds ratios (3.6, p. 14)
**Other analyses**	17	Report other analyses done—eg analyses of subgroups and interactions, and sensitivity analyses	3.5 Correlations, p. 13; Table 5, p. 13; 3.6 Sling Arm Morphology and Postoperative Dyspareunia, p. 14; Table 6, p. 14; correlation analysis adjusted for post-void residual volume, 4.6 Bladder Wall Thickness and Symptom Burden, p. 20
**DISCUSSION**			
**Key results**	18	Summarise key results with reference to study objectives	4.1 Overview, pp. 15–16
**Limitations**	19	Discuss limitations of the study, taking into account sources of potential bias or imprecision. Discuss both direction and magnitude of any potential bias	4.7 Strengths and Limitations, pp. 21–22: cross-sectional design and retrospective identification; absence of preoperative data; conservative dyspareunia denominator with stated direction of bias; no a priori power analysis; no correction for multiple comparisons; loss to contact; concomitant vaginal repair as a potential confounder; unassessed interobserver reliability; wide interval between surgery and assessment; bladder volume above the recommended measurement threshold; non-standardised bladder volume at stress testing; recall bias for postoperative OAB; single surgeon; single centre; no causal inference
**Interpretation**	20	Give a cautious overall interpretation of results considering objectives, limitations, multiplicity of analyses, results from similar studies, and other relevant evidence	4.2–4.6, pp. 16–21; 5. Conclusions, pp. 22–23
**Generalisability**	21	Discuss the generalisability (external validity) of the study results	4.7 Strengths and Limitations, p. 22; 5. Conclusions, p. 23 (findings limited to the outside-in transobturator sling)
**OTHER INFORMATION**			
**Funding**	22	Give the source of funding and the role of the funders for the present study and, if applicable, for the original study on which the present article is based	Funding, p. 24

**Give information separately for exposed and unexposed groups.*

Note: An Explanation and Elaboration article discusses each checklist item and gives methodological background and published examples of transparent reporting. The STROBE checklist is best used in conjunction with that article, which is freely available from the STROBE Initiative website (https://www.strobe-statement.org).
